# The Effect of COVID-19 on Male Sex Hormones: A Meta-Analysis of Prospective Cohort Study

**DOI:** 10.1007/s44197-024-00203-x

**Published:** 2024-02-26

**Authors:** Xiucheng Lan, Diang Chen, Meijing Wang, Xujun Yu, Liang Dong, Junjun Li, Degui Chang, Fang Yang

**Affiliations:** 1 Hospital of Chengdu University of Traditional Chinese Medicine, No. 39 shi-er-qiao Road, Chengdu, 610072 Sichuan Province China; 2 Chengdu University of Traditional Chinese Medicine, Chengdu, China; 3 Chengdu Fifth People’s Hospital, Chengdu, China

**Keywords:** COVID-19, Sex hormone, Testosterone, Luteinizing hormone, Male fertility

## Abstract

**Purpose:**

To evaluate the possible effects of novel coronavirus disease 2019 (2019-NCOV) on male sex hormones and reproductive ability, and analyze its incidence and risk factors.

**Methods:**

We retrieved from PubMed, Embase, The Cochrane Library, Web of Science, Clinical Trails, CNKI, CBM, Wan Fang Database and VIP to collect research on the effects of COVID-19 on the male sex hormone. Our literature search was conducted until April 2022, and two investigators independently screened articles based on inclusion and exclusion criteria. In strict accordance with the inclusion and exclusion criteria, two researchers independently screened the literature and comprehensively analyzed 8 cohort studies on the impact of COVID-19 on male sex hormone. And We used RevMan5.4.1 and Stata15.0 for statistical analysis. Finally, there were eight cohort studies on the effects of COVID-19 on male sex hormones.

**Results:**

T(RR = − 3.94; 95% CI − 6.22, − 1.66; P = 0.0007), testosterone in the COVID-19 group decreased by 3.94 nmol/L compared with the control group, and the difference was statistically significant. LH (RR = 0.85; 95% CI − 0.26, 1.96; P = 0.13), the LH in COVID-19 group was 0.85 mlU/ml higher than that in control group, but the difference was not statistically significant. FSH (RR = 0.25; 95% CI − 0.72, 1.23; P = 0.61), the FSH of COVID-19 group was 0.25 mlU/ml higher than that of the control group, but the difference was not statistically significant. PRL (RR = 2.42; 95% CI 0.52, 4.31; P = 0.01), the PRL in the COVID-19 group was 2.42 ng/ml higher than that in the control group, and the difference was statistically significant. E2(RR = 11.88; 95% CI 9.90, 13.86; P < 0.00001), The level of E2 in the COVID-19 group was 11.88 pg/ml higher than that in the control group, and the difference was statistically significant. T:LH (RR = − 0.39; 95% CI − 076, − 0.02; P = 0.04), the ratio of T:LH in COVID-19 group was lower than that in control group, and the difference was statistically significant. FSH:LH (RR = − 0.38; 95% CI − 0.86, 0.11; P = 0.13), the ratio of FSH:LH decreased in COVID-19 group compared with control group, but the difference was not statistically significant.

**Conclusions:**

COVID-19 can affect the level of sex hormones, especially T, which may further affect male fertility. Due to the limitations of this study, this conclusion needs to be further verified by large-sample, high-quality prospective cohort studies on the long-term effects of COVID-19 on male sex hormones and fertility.

## Introduction

Since December 2019, Corona Virus Disease 2019 (COVID-19) has erupted globally. As of April 24, 2022 (Beijing time), the number of confirmed COVID-19 cases worldwide has exceeded 440 million, and the cumulative number of deaths has exceeded 6 million. The domestic and foreign research shows that the effects of COVID-19 on male sex hormones are still controversial. It is believed that SARS-CoV-2 can cause substantial damage to male testicular tissue and induce changes in male sex hormone levels [1]. In the observational study by Apaydin et al. [2], most patients had low testosterone levels, and in the later 6-month follow-up, nearly half of patients still had testosterone levels below normal. Some patients’ testicles are infected with the SARS-CoV-2 virus, which is manifested by hypogonadism [3].

Testis produce sex hormones, and the normal physiological structure and function of testis are also the prerequisite for male reproduction. With the opening of the three-child policy, whether it will have an impact on male fertility is also an urgent concern of people. Given the potential impact and unknown outcomes of COVID-19 on the male reproductive system, it is necessary to determine reproductive outcomes in men with clinical symptoms or confirmed COVID-19. This study adoptedd the method of evidence-based medicine to collect published literature, objectively evaluate the impact of COVID-19 on sex hormones, and formulate corresponding defense measures to provide high-quality basis for primary prevention and health care services.

## Methods

### Scheme and Registration

Registered with PROSPERO as CRD42020181812.

### Search Strategy

PubMed, Embase, The Cochrane Library, Web of Science, Clinicaltrails, CNKI, CBM, Wanfang Database and VIP database were searched by computer. PubMed, Embase, The Cochrane Library, Web of Science, Clinicaltrails, CNKI, CBM, Wanfang Database and VIP database were searched by computer. All database literature is from inception to April 2022. Using the combination of free words and subject words, the search language is not limited. Search words include: SARS-CoV-2, COVID-19, Coronavirus Disease 2019 Virus, 2019 Novel Coronaviruses, Gonadal Hormones, male sex hormones, testosterone, luteinizing hormone, Follicle Stimulating Hormone, male reproductive system, etc.

### Inclusion and Exclusion Criteria

Inclusion criteria: (1) Published literature or ongoing clinical trials data; (2) Cohort studies or case–control studies with COVID-19 and control groups, whether or not double-blind; (3) Patients in the experimental group were at the stage of COVID-19 infection or recovery. The control group was non-COVID-19 normal healthy people; (4) The variable detection methods in literature were consistent.

Exclusion criteria: (1) No control group or poor balance between groups, no comparability; (2) The research contents are inconsistent or do not meet the inclusion criteria; (3) Cross-sectional studies, reviews, experience summaries, theoretical discussions, experimental animal studies, case reports, and repeated published studies; (4) The experimental group or the control group had infections other than COVID-19, drugs affecting the HPA axis, hypothalamic-pituitary diseases treated with hormone therapy, chemotherapy with immunosuppressive drugs, etc. (5) The full text cannot be obtained or the outcome indicators are inconsistent; (6) No valid data were extracted or there were obvious errors in the data.

### Literature Quality Assessment

The Newcastle–Ottawa Scale (NOS) was used to evaluate the quality of the literature of prospective cohort studies, which mainly consisted of the following three aspects: (1) The selection of the study population (4 points): ①whether the exposure group is representative: 1 point from a random sample of the general population, no points from special groups, such as nurses, volunteers, etc.; ② Selection of non-exposed group: 1 point for being from the same population as the exposed group, otherwise no points; ③ Confirmation of exposure: 1 point for patients diagnosed with COVID-19 by definite nasopharyngeal swabs or oral specimens receive; otherwise no points; ④ 1 point for absence of urinary tract disease and COVID-19 symptoms prior to follow-up, otherwise no points. (2) Comparability between groups (2 points): whether the relevant confounding factors were adjusted or not: 2 points for adjusting age and important confounding factors related to the disease, 1 point for adjusting age or important confounding factors related to the disease, and no points for not adjusting either. (3) Result evaluation (3 points): ① Evaluation of outcome: 1 point for clear recording of sex hormone data, otherwise no score; ② Follow-up years: 1 point for more than 60 days of follow-up, otherwise no points; ③ Cohort population lost visit rate: less than 25% scored 1 point, otherwise no score. The results of the literature scoring are shown in Table 2. NOS scores out of 9, with 0 to 3, 4 to 6, and 7 to 9 being low, medium, and high quality studies in that order.

### Literature Screening and Data Extraction

The data will be collected and screened by two assessors independently. In case of divergence, it will be resolved through discussion. If necessary, a third researcher will make the judgment. The extracted content includes the publication year, the first author, the research center, the total number of patients in the control group and the experimental group, the average age, clinical symptoms, BMI (Body Mass Index), and outcome indicators. After data extraction, the third researcher will check the extracted results. If there is any discrepancy in the data, it will be processed through group discussion or consultation with professional statisticians. In addition, for the analysis of sex hormone levels, we will collect the following data: testosterone (T, nmol/L), luteinizing hormone (LH, mlU/ml), follicle-stimulating hormone (FSH, mlU/ml), prolactin (PRL, ng/ml), estradiol (E2, pg/ml).

### Statistical Methods

We will use RevMan 5.4.1 tool to draw forest plot, and use Stata17.0 software to generate funnel plot and Egger’s test. When evaluating heterogeneity among studies, we will adopt Q value and I^2^ value. When I^2^ ≤ 50% or P ≥ 0.1, it indicates no significant heterogeneity. Subsequently, we will calculate the P value, RR value and its 95% confidence interval of the combined statistics. To further evaluate publication bias, we will use funnel plot and Egger’s test. If the P ≥ 0.05, it indicates no significant publication bias. For data that cannot be processed by meta-analysis, we will use descriptive statistics for analysis.

## Results

### Screening Process and Eligible Studies

**Fig. 1:**
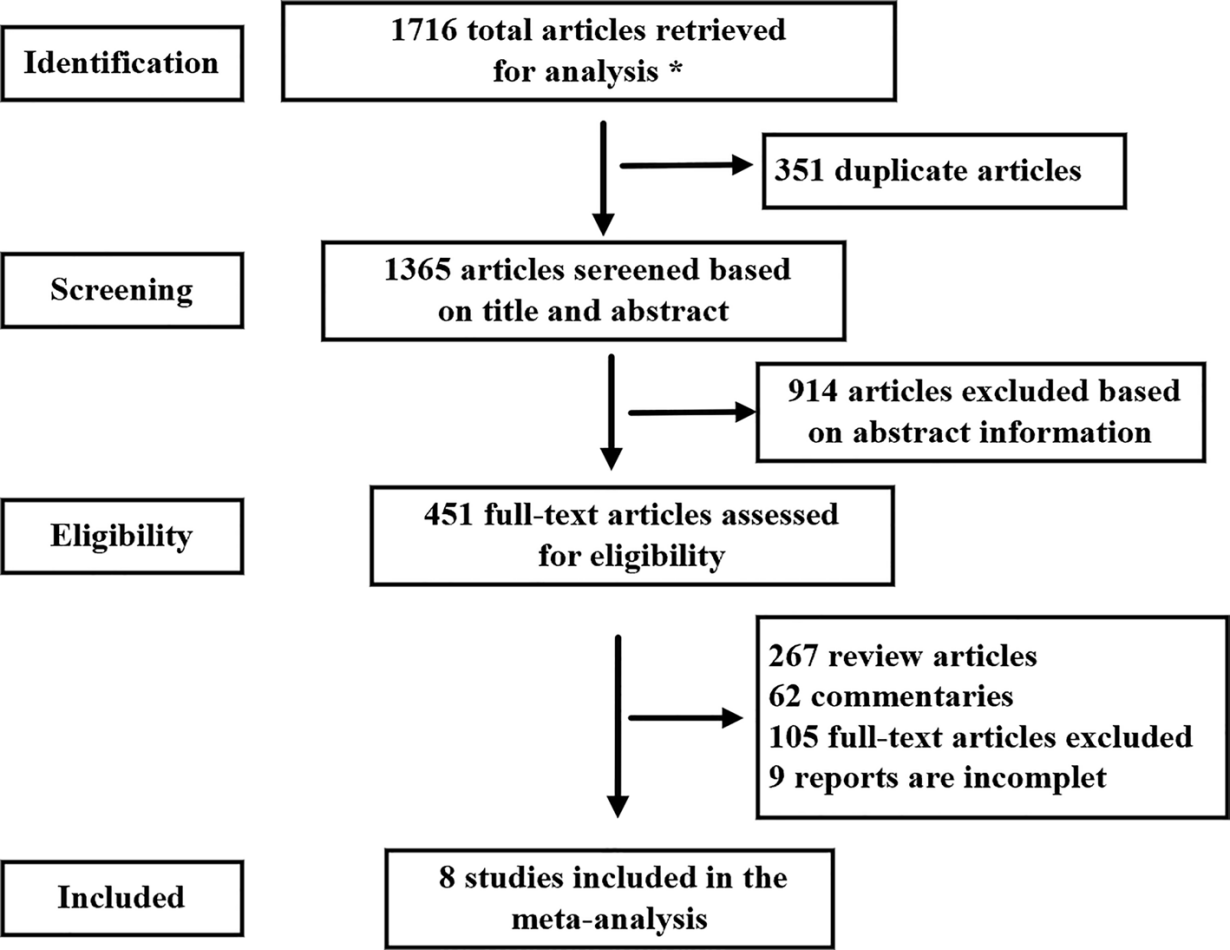
Summary of the literature identification and selection process. ∗ signifies PubMed = 87, Cochrane library = 27, Embase = 1054, Web of science = 506, CBM = 6, CNKI = 4, Wanfang = 22, VIP = 10

A total of 1716 studies were included. After reading the title, abstract and full text, and excluding the articles that did not meet the inclusion criteria, 8 articles were finally included, all of which were prospective cohort studies in English. The literature screening process and results are shown in Fig. 1.

### Study Characteristics

A total of eight studies were included in this meta-analysis, including three from China, one from the Russia, two from Turkey, and the other two are not recorded. A summary of the included studies is presented in Table 1. (The experimental group is in the front, and the healthy group is in the back).

**Table 1:** Basic characteristics of the included literature

References	Study location	Study design	Number	Age	BMI	Outcome index
Cinislioglu et al. [4]	Turkey	Prospective cohort study	358:92	64.9 ± 11.6 67.2 ± 13.6	25.9 ± 3.8 26.4 ± 3.1	①②③⑤
Enikeev et al. [5]	Russia	Prospective cohort study	44:44	46.7 ± 9.9 30.7 ± 9.8	NA	①②③④
Guo et al. [6]	China	Prospective cohort study	41:50	26 ± 1.48 26.5 ± 6.67	25.1 ± 4.51 NA	①②③④
Kadihasanog et al. [7]	NA	Prospective cohort study	89:143	49.9 ± 12.5 50 ± 7.8	NA	①②③④
Ma et al. [8]	China	Prospective cohort study	119:273	39 ± 6.66 39 ± 5.18	NA	①②③⑥⑦
Salonia et al. [9]	NA	Prospective cohort study	286:281	58 ± 12.59 46 ± 12.59	27.8 ± 4.22 25.1 ± 3.11	①②③
Temiz et al. [10]	Turkey	Prospective cohort study	10:10	38 ± 8.28 36.6 ± 9.63	25.55 ± 2.08 26.57 ± 2.71	①②③④⑥⑦
Xu et al. [11]	China	Prospective cohort study	39:22	60 ± 14.07 62 ± 12.4	25.1 ± 2.8 26.9 ± 3.6	①②③④⑤

*BMI* Body Mass Index, *NA* not applicable

① T; ② LH; ③ FSH; ④ PRL; ⑤ E2; ⑥ T: LH; ⑦ T: LH

### Quality Evaluation of the Included Studies

The included articles were all prospective cohort studies, and their risk of bias was evaluated according to the NOS scale. Eight of the included literatures had the study score of ≥ 6 stars, and the quality of the included studies was high. The specific scores are shown in Table 2.

**Table 2:** NOS scores for the included studies

References	Selection	Comparability	Outcome	Total score
Cinislioglu et al. [4]	☆☆☆	☆☆	☆	Six stars
Enikeev et al. [5]	☆☆☆☆	☆☆	☆	Seven stars
Guo et al. [6]	☆☆☆☆	☆☆	☆☆	Eight stars
Kadihasanog et al. [7]	☆☆☆☆	☆☆	☆	Seven stars
Ma et al. [8]	☆☆☆	☆☆	☆	Six stars
Salonia et al. [9]	☆☆☆☆	☆☆	☆	Seven stars
Temiz et al. [10]	☆☆☆☆	☆☆	☆	Seven stars
Xu et al. [11]	☆☆☆	☆☆	☆	Six stars

## Results of Meta-Analysis on Sex Hormones

### Testosterone

**Fig. 2:**
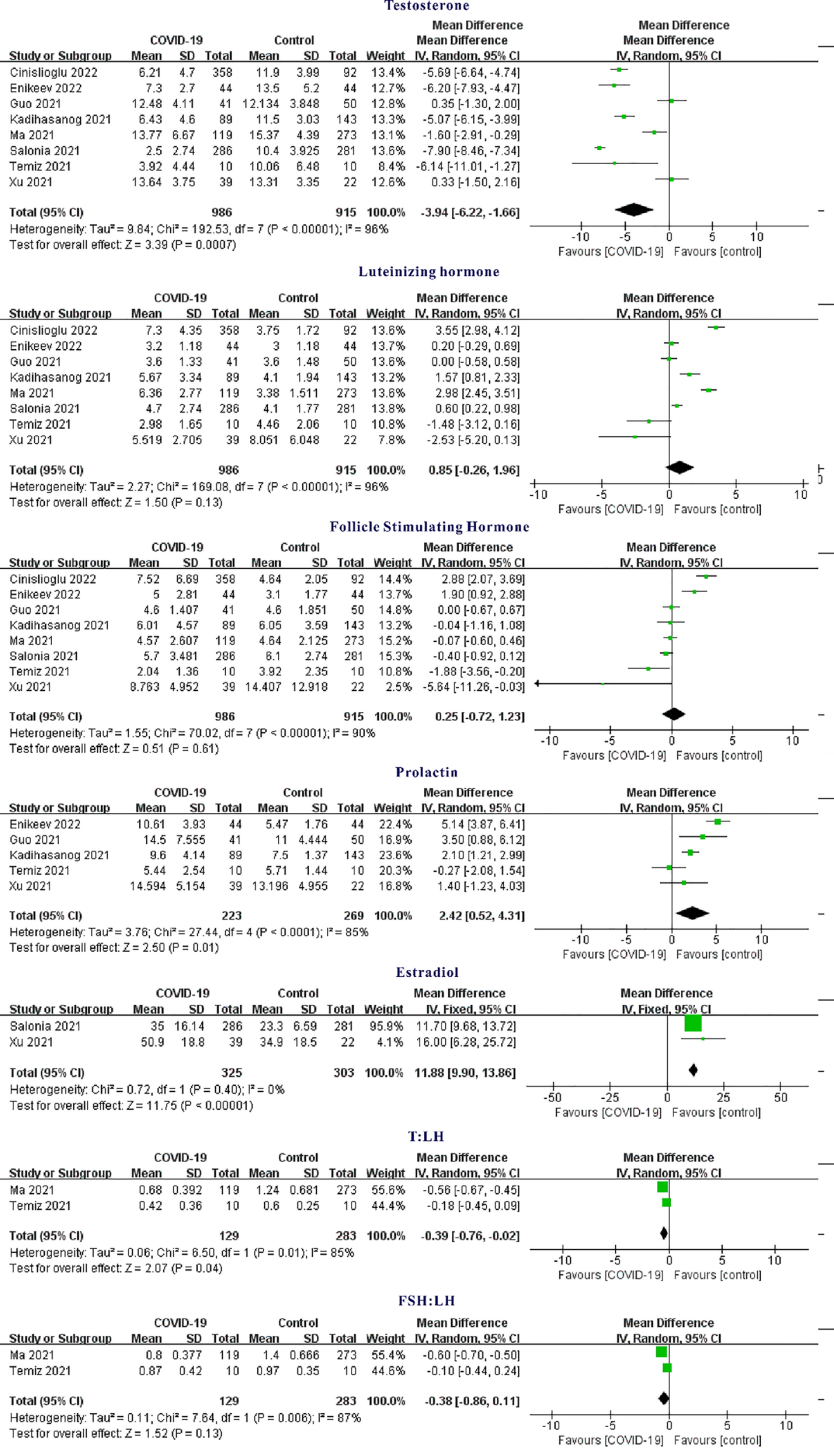
Forest plot of sex hormones between COVID-19 group and healthy control group

There are 8 articles about the effect of COVID-19 on testosterone [4–11], and testosterone was 3.94 nmol/L lower in the COVID-19 group than in the healthy control group (overall MD − 3.94; 95% CI − 6.22, − 1.66; Z = 3.39, P = 0.0007) (Fig. 2). However, MDs between-study were highly varied (I^2^ = 96%, P < 0.00001).

### Luteinizing Hormone

According to the meta-analysis from 8 [4–11], Luteinizing hormone of those subjects COVID-19 was 0.85 more than those healthy control group (overall MD: 0.85; 95% CI − 0.26, 1.96; Z = 1.50, P = 0.13 > 0.05) (Fig. 2), but the difference was not statistically significant.

### Follicle Stimulating Hormone

Meta-analysis from 8 studies [4–11] revealed that Follicle Stimulating Hormone COVID-19 was 0.25 mlU/ml more than those healthy control group (overall MD 0.25; 95% CI − 0.72, 1.23; Z = 0.51, P = 0.61 > 0.05) (Fig. 2). However, its MD was highly varied.

### Prolactin

MD from 5 studies [5–7, 10, 11] about Prolactin were highly varied (I^2^ = 85%, P = 0.0001). There was statistically significant pooled MD (95% CI) of 2.42 (0.52, 4.31) (Z = 2.50, P = 0.01) suggesting that the prolactin of those COVID-19 group was 2.42 ng/ml higher than these healthy control group (Fig. 2).

### Estradiol

According to two studies [4, 11], estradiol level among those COVID-19 group was more than that among healthy control groups with overall MD (95% CI) of 11.88 (9.90, 13.86) (Z = 11.75, P < 0.00001) with presence of low heterogeneity (I^2^ = 0%, P = 0.4).

### T: LH

T: LH among those COVID-19 group is less than that in healthy control group overall MD − 0.39, 95% CI (− 076, − 0.02), Z = 2.07, P = 0.04 (Fig. 2). MD between these 2 studies [8, 10] were highly varied (I^2^ = 85%, P = 0.1).

### FSH: LH

MD about FSH: LH from 2 studies [8, 10] were highly varied (I^2^ = 87%, P = 0.006) (Fig. 2). The results showed that the COVID-19 group was 0.38 lower than the healthy control group, but the difference was not statistically significant. (Overall MD: − 0.38; 95% CI – 0.86, 0.11; Z = 1.52, P = 0.13 > 0.05).

## Sensitivity Analysis and Subgroup Analysis

**Fig. 3:**
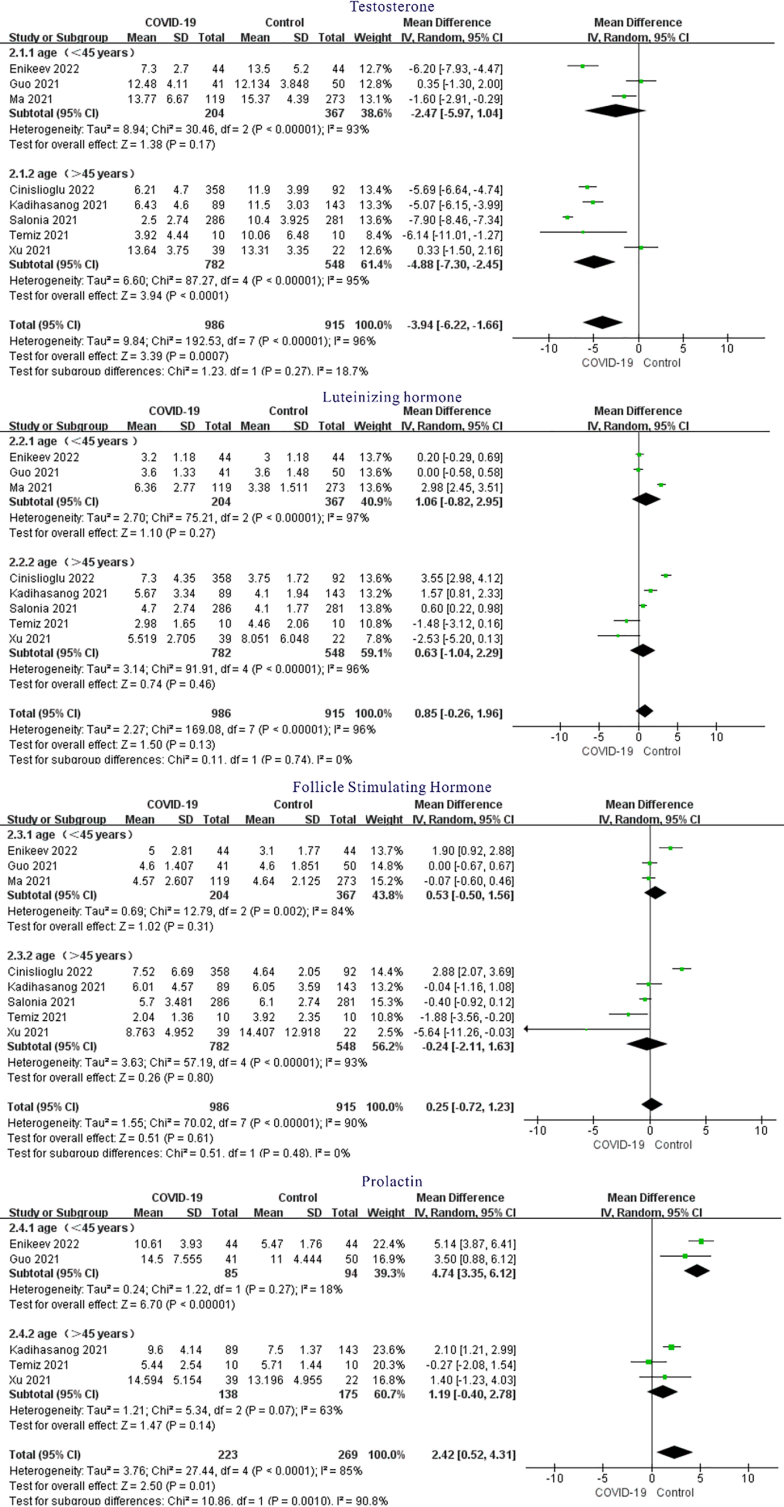
Forest plot of sex hormone subgroup analysis between COVID-19 group and healthy control group

As analyzed above, there was great heterogeneity among studies. When the literature was removed one by one for sensitivity analysis, the pooled results of the remaining studies did not change significantly, indicating that the research results were relatively robust. We considered that the heterogeneity might be caused by differences in age and clinical characteristics. When we conducted subgroup analysis based on age differences, we found that the heterogeneity among subgroups was still large, and the differences in the combined results were different, indicating that age may not be the source of the differences (Fig. 3). Due to the diversity of clinical features, it is impossible to carry out subgroup analysis, which may be one of the sources of differences.

## Test of Publication Bias

**Fig. 4:**
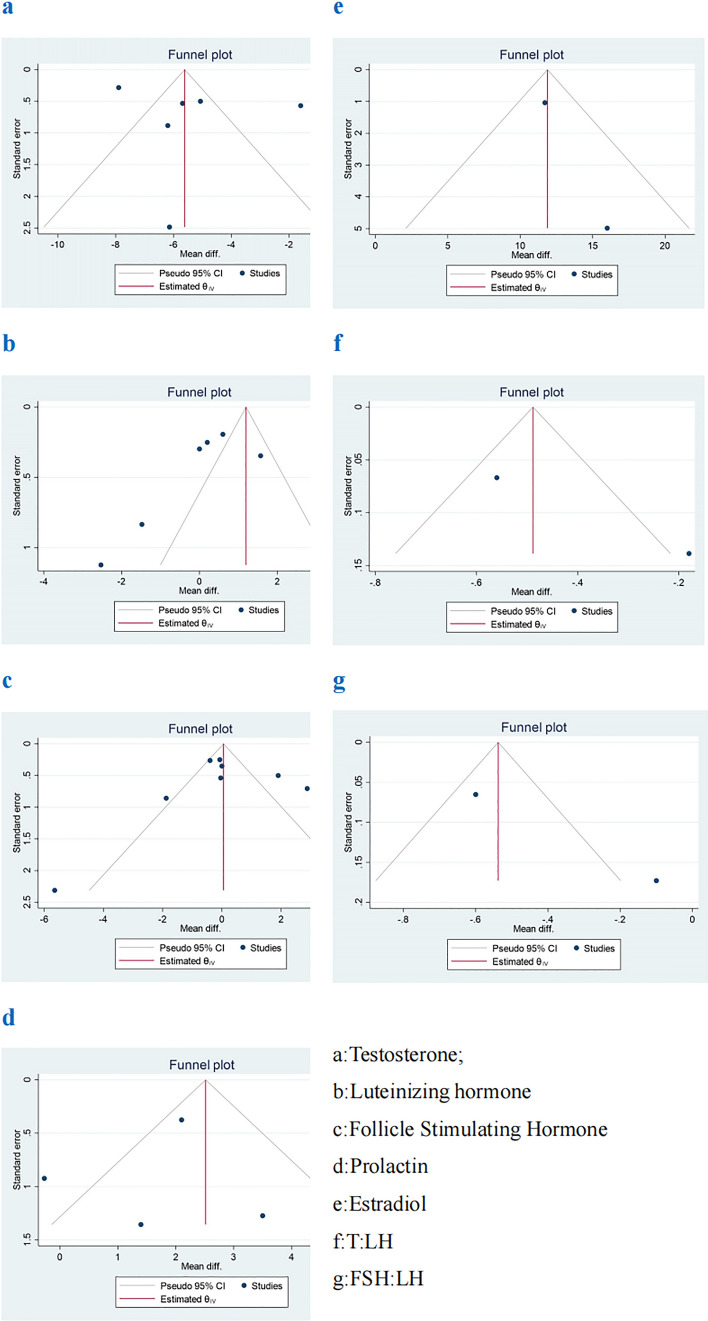
Funnel plot of sex hormones between COVID-19 group and healthy control group

In this systematic review, we simultaneously used funnel plot and Egger’s test to analyze publication bias of articles. The funnel plots in most studies are asymmetric and uneven (Fig. 4). The reason may be that the number of eligible studies in the meta-analysis in this study is relatively small and the research index are different, which may lead to a further reduction in the number of included studies. At the same time, some sample sizes of some articles are small, and there are regional differences and heterogeneity.

**Fig. 5:**
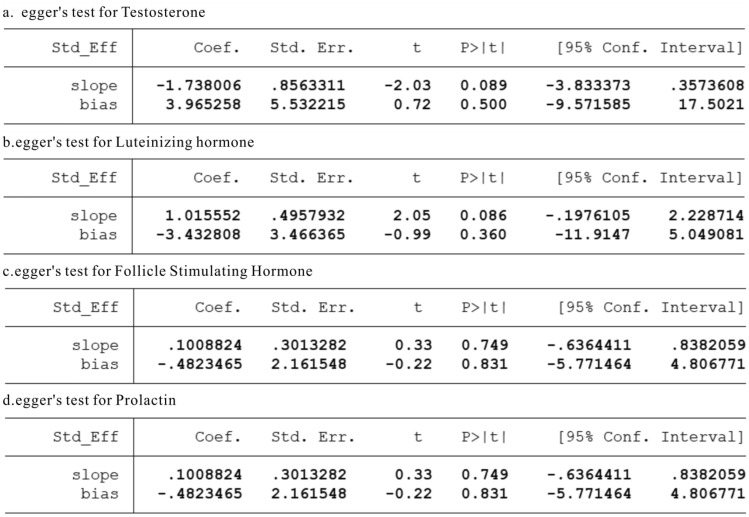
Egger’s test of sex hormones between COVID-19 group and healthy control group

However, Egger’s test showed that there was no publication bias between the COVID-19 group and the healthy control group, and the results were as follows. T: coefficient = 3.965, SE = 5.53, P = 0.5 > 0.05 (Fig. 5a). LH: coefficient = − 3.432, SE = 3.466, P = 0.36 > 0.05 (Fig. 5b). FSH: coefficient = − 0.482, SE = 2.161, P = 0.831 > 0.05 (Fig. 5c). PRL: coefficient = − 1.449, SE = 3.376, P = 0.697 > 0.05(Fig. 5d). There are three studies such as E2, T: LH and FSH: LH included too few literatures to make Egger’s test chart.

## Discussion

Sex hormones and the ratio of sex hormones are closely related to semen quality, sperm fertilization ability and fertility [12, 13]. And low levels of sex hormones may lead to increased sperm DNA damage, further increasing the possibility of male infertility [14]. The invasion of SARS-CoV-2 is mainly related to human angiotensin converting enzyme 2 (ACE2) and type II transmembrane serine protease (TMPRSS2). Androgens are mainly synthesized in interstitial cells, and ACE2 and TMPRSS2 are expressed in interstitial cells [15, 16]. Study has shown that testosterone can regulate the expression of ACE2 and TMPRSS2 simultaneously [17], which may promote the internalization of SARS-CoV-2 [18]. And compared to estrogen, testosterone may make men more susceptible to COVID-19 than women [18, 19].

Since basal T levels may vary considerably in populations, the ratio between hormones such as T: LH and FSH: LH is considered to be a better parameter for assessing male gonadal function [8]. The decrease of T level and T:LH ratio in this study indicates that COVID-19 will affect the level of male sex hormones, leading to hypogonadism and even infertility [2, 20]. There are different views on the mechanism of COVID-19 affecting T level. Sengupta et al. believe that SARS-CoV-2 damages leydig cells, resulting in the synthesis of T and other hormones [21]. However, Adel believed that SARS-CoV-2 directly affected the production of sex hormones and the hypothalamic-pituitary–testicular axis, ultimately leading to primary leydig cell injury [22]. The mechanism by which COVID-19 affects hormone levels remains to be further identified.

We analyzed the reasons for the high heterogeneity of this systematic review. First, BMI has an effect on male hormones [23, 24], but at the time of inclusion in this literature, some samples were not well informed about BMI and therefore did not perform subgroup analysis. Secondly, the synthesis of hormones is mainly in the testis. In addition to direct effects of SARS-CoV-2 virus on testis, fever, inflammation and other factors related to immune and stress response are also easily involved in the synthesis of testosterone. All the eligible studies included had fever symptoms, but there was no difference in fever grade, so subgroup analysis could not be performed. However, the diversity and uncertainty of clinical features, as well as whether various clinical manifestations will influence each other, also make subgroup analysis impossible. Finally, when we pooled the data by age group, the results showed that age was not the source of heterogeneity, suggesting that the effects of COVID-19 on male sex hormones were independent of age. Therefore, compared with non-reproductive age, male patients with fertility requirements and childbearing age should pay more attention to sex hormone levels, actively evaluate and consult, and prepare for the normalization of the epidemic.

There are also limitations to this systematic review. First of all, the sample size of the included studies varied greatly among studies, which may affect the accuracy of the meta-analysis results. Secondly, the search was limited to Chinese and English, and the literature may not be comprehensive enough to miss relevant studies in other languages. Finally, there were no long-term follow-up data from all studies to determine whether SARS-CoV-2 would have a long-term effect on sex hormones.

## Conclusion

In summary, COVID-19 has a direct impact on sex hormones and may threaten male fertility, but the long-term effects on patients and whether they are reversible are unknown, and follow-up needs to be extended. More high-quality, large-sample studies need to be included in the future to further reveal the effects of COVID-19 on male sex hormones.

## Data Availability

The authors vouch for the authenticity of the data and materials.

## Abbreviations

COVID-19: Corona Virus Disease 2019
BMI: Body mass index
LH: Luteinizing hormone
FSH: Follicle stimulating hormone
PRL: Prolactin
E2: Estradiol
ACE2: Angiotensin converting enzyme 2
TMPRSS2: Type II transmembrane serine protease
